# *Arthrospira platensis* var. *toliarensis*: A Local Sustainable Microalga for Food System Resilience

**DOI:** 10.3390/foods14152634

**Published:** 2025-07-27

**Authors:** Antonio Fidinirina Telesphore, Andreea Veronica Botezatu, Daniela Ionela Istrati, Bianca Furdui, Rodica Mihaela Dinica, Valérie Lalao Andriamanamisata Razafindratovo

**Affiliations:** 1 Département Biochimie Fondamentale et Appliquée–Sciences des Aliments et Nutritrion, Domaine Sciences et Technologies, Université d’Antananarivo, Antananarivo 101, Madagascar; fidinirina.telesphore@gmail.com (A.F.T.); lalaovalerie@yahoo.fr (V.L.A.R.); 2 Laboratoire de Biochimie Appliquée aux Sciences de l’Alimentation et à la Nutrition (LABASAN), Université d’Antananarivo, Antananarivo 101, Madagascar; 3 Faculty of Sciences and Environment, Department of Chemistry, Physics and Environment, “Dunarea de Jos” University of Galati, 111 Domneasca Street, 800201 Galati, Romania; andreea.botezatu@ugal.ro (A.V.B.); rodica.dinica@ugal.ro (R.M.D.); 4 Faculty of Food Science and Engineering, “Dunarea de Jos” University of Galati, 111 Domneasca Street, 800201 Galati, Romania; daniela.istrati@ugal.ro

**Keywords:** *Arthrospira platensis* var. *toliarensis*, local microalgae, protein productivity, essential amino acids, antioxidant activity, phenolic compounds, mineral composition, superfood, sustainable food systems

## Abstract

The intensifying global demand for sustainable and nutrient-dense food sources necessitates the exploration of underutilized local resources. *Arthrospira platensis* var. *toliarensis*, a cyanobacterium endemic to Madagascar, was evaluated for its nutritional, functional, and environmental potential under small-scale, low-input outdoor cultivation. The study assessed growth kinetics, physicochemical parameters, and composition during two contrasting seasons. Biomass increased 7.5-fold in 10 days, reaching a productivity of 7.8 ± 0.58 g/m^2^/day and a protein yield of 4.68 ± 0.35 g/m^2^/day. The hot-season harvest showed significantly higher protein content (65.1% vs. 44.6%), enriched in essential amino acids. On a dry matter basis, mineral profiling revealed high levels of sodium (2140 ± 35.4 mg/100 g), potassium (1530 ± 21.8 mg/100 g), calcium (968 ± 15.1 mg/100 g), phosphorus (815 ± 13.2 mg/100 g), magnesium (389.28 ± 6.4 mg/100 g), and iron (235 ± 9.1 mg/100 g), underscoring its value as a micronutrient-rich supplement. The hydroethanolic extract had the highest polyphenol content (4.67 g GAE/100 g of dry extract), while the hexanic extract exhibited the strongest antioxidant capacity (IC_50_ = 101.03 ± 1.37 µg/mL), indicating fat-soluble antioxidants. Aflatoxin levels (B1, B2, G1, and G2) remained below EU safety thresholds. Compared to soy and beef, this strain showed superior protein productivity and water-use efficiency. These findings confirm *A. platensis* var. *toliarensis* as a promising, ecologically sound alternative for improving food and nutrition security, and its local production can offer substantial benefits to smallholder livelihoods.

## 1. Introduction

Global food security, undermined by the cumulative effects of climate change, natural resource degradation, and rapid urbanization, remains a structural challenge, particularly in the Global South. In 2023, over 733 million people suffered from hunger, a steadily rising figure since 2015 [1]. This nutritional insecurity is especially severe in developing countries, such as Madagascar, where more than 40% of children under five suffer from chronic malnutrition linked to structural poverty and agricultural instability [2,3].

A key underlying factor in this crisis lies in the disproportionate reliance of the global food supply on a limited pool of cultivated crops. It is estimated that just 103 plant species are responsible for delivering nearly 90% of the world’s caloric intake, with rice, wheat, and maize alone contributing close to 60% [4,5]. Similarly, protein consumption at the global scale is largely sourced from a handful of intensively farmed species, mostly cereals and legumes, which jointly contribute 57% of protein intake, supplemented by animal-based products like meat (18%), dairy (10%), and fish (6%) [6,7]. This narrow base of production undermines agroecosystem diversity, exposes food systems to environmental shocks, and restricts access to balanced nutrition [8].

In light of these challenges, diversifying food sources with options that are both nutritionally rich and environmentally sound has become increasingly vital, especially in regions facing chronic food insecurity. Among these alternatives, microalgae, and, in particular, members of the *Arthrospira genus*, have attracted growing interest due to their dense nutrient content, high productivity, minimal ecological impact, and adaptability across diverse cultivation environments [9,10]. Nevertheless, research efforts have predominantly focused on standardized commercial strains grown in controlled or intensive settings, leaving many native and locally adapted varieties underexplored. Among the most extensively studied microalgae are *Chlorella vulgaris* and commercial *Arthrospira platensis* strains, which are typically optimized in closed or semi-controlled systems to maximize yield and purity. *Chlorella vulgaris*, in particular, requires tight control of temperature, light intensity, and pH stability, as deviations can lead to reduced growth and productivity [11]. In contrast, *A. platensis* var. *toliarensis* thrives in naturally alkaline, open environments with limited infrastructure, offering a resilient and low-input alternative.

*Arthrospira platensis* var. *toliarensis* is an endemic strain from southwestern Madagascar, cultivated by small-scale producers using artisanal methods. Despite its promising nutritional and ecological attributes, this microalga remains scarcely represented in global scientific discourse. It highlights the untapped biological resources of tropical regions and prompts reflection on the role of neglected species in shaping future food security strategies.

This research seeks to fill the knowledge gap by providing a thorough assessment of the nutritional composition, antioxidant activity, potential toxicological risks, and environmental performance of *A. platensis* var. *toliarensis* under semi-artisanal cultivation conditions, defined as small-scale, low-input systems using open-door tanks, with partially controlled parameters, such as salinity and pH, but without mechanized equipment or automation. The study also examines how seasonal variations, comparing cooler and warmer periods, affect its biomass yield and biochemical traits. Framed within a broader sustainability agenda, this work aims to elevate this local cyanobacterium as a viable component of resilient, context-specific food systems.

## 2. Materials and Methods

### 2.1. Presentation of the Strain and Sampling

The biological material investigated in this study is a filamentous cyanobacterium, *Arthrospira platensis* var. *toliarensis*, locally known as Malagasy spirulina. First identified by Fox in 1986 and taxonomically confirmed in the 1990s, this strain is endemic to the alkaline mineral-rich basins of Toliara, southwest Madagascar [12,13]. Cultivated semi-industrially by SPIRISUD in Maninday, this strain is notable for its resilience to arid climatic conditions, high solar radiation, temperature, and salinity [14].

### 2.2. Experimental Cultivation Procedures

#### 2.2.1. Experimental Design and Culture Conditions

**Figure 1:**
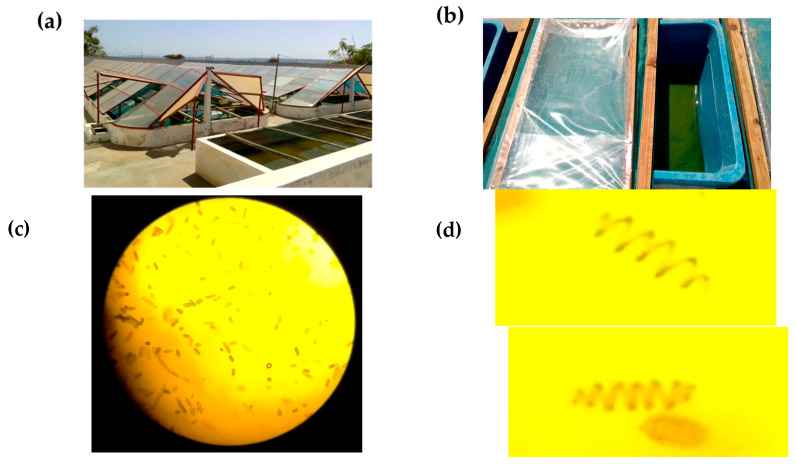
(**a**) Semi-industrial raceway ponds of SPIRISUD; (**b**) plastic tanks (dimensions: 50 × 25 × 30 cm) for small-scale artisanal cultivation; (**c**) light microscopy images of *A. platensis* var. *toliarensis* (100×); (**d**) zoomed-in view of (**c**), showing detailed helical trichomes of *A. platensis* var. *toliarensis*.

The growth performance of *Arthrospira platensis* var. *toliarensis* was monitored over 10 days under semi-controlled natural conditions using artisanal infrastructure to simulate accessible methods for small-scale producers (Figure 1). Open plastic tanks (dimensions: 50 × 25 × 30 cm) were used and installed outdoors under natural sunlight. Three (3) tanks were used to triplicate the experiment. Each tank contained 25 L of culture mixture, composed of 5 L of concentrated inoculum (0.3 g/L dry weight; turbidity corresponding to a Secchi depth of 3 cm) and 20 L of nutrient medium prepared following Jourdan’s formulation [15]: NaHCO_3_ (8 g/L), NaCl (5 g/L), KNO_3_ (2 g/L), K_2_SO_4_ (1 g/L), NH_4_H_2_PO_4_ (0.2 g/L), MgSO_4_ 7H_2_O (0.2 g/L), CaCl_2_ 2H_2_O (0.1 g/L), and urea (0.01 g/L). The medium was prepared using distilled and sterilized water. Although no aseptic techniques or microbial testing were performed, the culture medium was prepared under clean conditions, and the tanks were frequently covered to minimize the risk of contamination.

**Figure 2:**
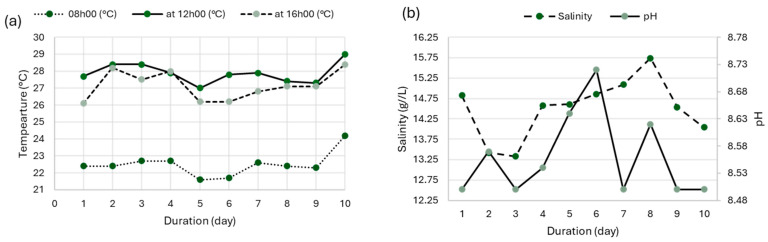
(**a**) Variation in temperature as a function of culture time; (**b**) variation in salinity as a function of culture time.

The initial biomass concentration was 0.06 g/L, corresponding to a Secchi disk visibility depth of approximately 6–7 cm. The initial pH was adjusted to 8.5 using sodium bicarbonate and maintained between 8.5 and 10 throughout the experiment using daily monitoring with a portable pH meter. Salinity was initially adjusted to 13 g/L and maintained between 13 and 16 g/L by compensating evaporative losses with deionized water. Measurements were performed daily using a handheld refractometer (KER-ORA1SA, Kern & Sohn GmbH, Balingen, Germany). Temperature was recorded three times daily (08:00, 12:00, and 16:00) with a digital thermometer (Figure 2). Manual stirring was carried out twice per day to ensure homogeneity and prevent sedimentation. No additional nutrients were added during the 10-day experiment.

#### 2.2.2. Growth Monitoring and Biomass Estimation

Daily growth was assessed through turbidity measurements using a 5 cm diameter Secchi disk mounted on a calibrated rod, as described by Gilles and Charito [16]. A calibration curve previously established in preliminary experiments was used to correlate Secchi depth with dry biomass concentration (g/L), allowing non-destructive and rapid monitoring. The calibration curve was established by measuring the dry weight (dried at 105 °C until constant weight) of samples corresponding to different Secchi depths, allowing accurate estimation of biomass from turbidity readings. In addition to turbidity, microscopic observations were conducted on days 0, 4, 7, and 10 using 10 µL samples mounted on microscope slides. Morphological characteristics, such as filament entanglement, trichome length, and cell density, were recorded. The specific growth rate (*μ*), dry biomass yield, and surface productivity (SP) were also evaluated by the following formulas:

$$\mu \;=\frac{ln\left(C_{t}\right)-ln(C_{initial})}{t}$$

$$Dry\;biomass\;yield\;\left(g\right)=(C_{final}-C_{initial})\times V$$

$$Surface\;productivity\;(g/m^{2}/day)=\frac{Dry\;biomass\;yiels}{Surface\;area\times duration}$$

where C_initial_ is the initial biomass concentration (g/L), C_t_ is the concentration at time (in days), C_final_ is the final concentration, and V is the total culture volume (25 L). The tank surface area was 0.125 m^2^, and the duration of cultivation was 10 days.

### 2.3. Sample Collection and Biochemical Analyses

#### 2.3.1. Sampling and Preparation

Biomass of *Arthrospira platensis* var. *toliarensis* was collected from open raceway ponds managed by SPIRISUD, a local producer located in Maninday, Toliara, Madagascar (23°19′35.3″ S, 43°42′2.9″ E). To evaluate the effect of seasonal variation on biomass composition, two distinct harvest periods were selected: October (representing the hot season) and May (representing the cool season). In both cases, the biomass was harvested using standard procedures established by SPIRISUD. After manual collection, the biomass was filtered, rinsed with clean water to remove debris and salts, mechanically pressed to remove excess moisture, and then dried at low temperature (<45 °C) to preserve nutritional quality. The dried material was subsequently ground into a fine powder using a stainless-steel grinder and stored in hermetically sealed packaging at an ambient temperature in the dark until analysis.

#### 2.3.2. Physicochemical and Nutritional Composition

The proximate composition of dried biomass, including moisture, ash, crude protein, lipids, and carbohydrates, was determined following standardized methods from the *AOAC Official Methods of Analysis*, 21st edition (2019) [17]. Moisture content was determined according to AOAC 925.10. Approximately 5.00 ± 0.01 g of homogenized sample was weighed into a pre-dried crucible and placed in a convection oven at 105 °C for 24 h. The samples were then cooled in a desiccator for 30 min, and the weight loss was recorded to calculate the moisture percentage on a wet basis. Ash content was analyzed using AOAC 923.03. About 5.00 ± 0.01 g of the dried sample was placed in a pre-weighed porcelain crucible and incinerated in a muffle furnace at 685 °C for 6 h, until the sample turned into a light gray ash. The crucibles were cooled in a desiccator before final weighing to determine the mineral residue. Crude protein was quantified using the Kjeldahl method (AOAC 2001.11). Samples were digested with concentrated sulfuric acid. The digested solution was neutralized with sodium hydroxide and distilled using steam distillation. The released ammonia was collected in a boric acid solution and titrated with standardized hydrochloric acid. Nitrogen content was converted to protein using the standard factor 6.25. Total lipids were extracted using the Soxhlet method (AOAC 991.36) with hexane as the solvent. Approximately 5.00 ± 0.01 g of the dried and ground sample was placed in a cellulose thimble and extracted for 6 h. After extraction, the solvent was evaporated, and the residue was dried and weighed to obtain the gravimetric lipid content. Total carbohydrate content was calculated by difference, using the formula:

$$Carbohydrates\;\left(\%\right)=100-[Moisture(\%)\;+\;Ash(\%)\;+Protein(\%)\;+\;Lipids(\%)]$$

#### 2.3.3. Amino Acid Analysis by HPLC-UV

The amino acid profile of *Arthrospira platensis* var. *toliarensis* powder was analyzed according to ISO 13903:2005 [18]. This procedure involves acid hydrolysis of proteins to release their constituent amino acids, followed by derivatization and high-performance liquid chromatography (HPLC) analysis. Separation was achieved on a dedicated reversed-phase column, and quantification was performed using UV detection on HPLC System (Waters, Milford, MA, USA).

Proteins were hydrolyzed using 6 N HCl at 110 °C for 24 h under nitrogen to prevent oxidation. After cooling, the hydrolysate was filtered, neutralized with 6 N NaOH to pH 6.5–7.0, and diluted to 25 mL with ultrapure water. Tryptophan was excluded due to degradation during acid hydrolysis.

A 1 mL aliquot was derivatized using the AccQ•Tag™ reagent (Waters, Milford, MA, USA) containing 6-aminoquinolyl-N-hydroxysuccinimidyl carbamate (AQC) and then incubated at 55 °C for 10 min. Separation of AQC-derivatized amino acids was performed using a Waters AccQTag™ column (3.9 × 150 mm), and detection was carried out by UV at 254 nm. For sulfur-containing amino acids (cysteine, cystine, and methionine), pre-oxidation was performed with performic acid (freshly prepared from formic acid and hydrogen peroxide). A 200 µL aliquot was added to 50 mg of the sample and incubated at 0–4 °C for 16 h, converting the compounds to stable cysteic acid and methionine sulfone. The oxidized sample was then processed, as above. Quantification was based on calibration with certified amino acid standards (Sigma-Aldrich, St. Louis, MO, USA), and the results are expressed as mg/100 g of dry matter (DM).

#### 2.3.4. Mineral Element Quantification by ICP-OES and ICP-MS

**Figure 3:**
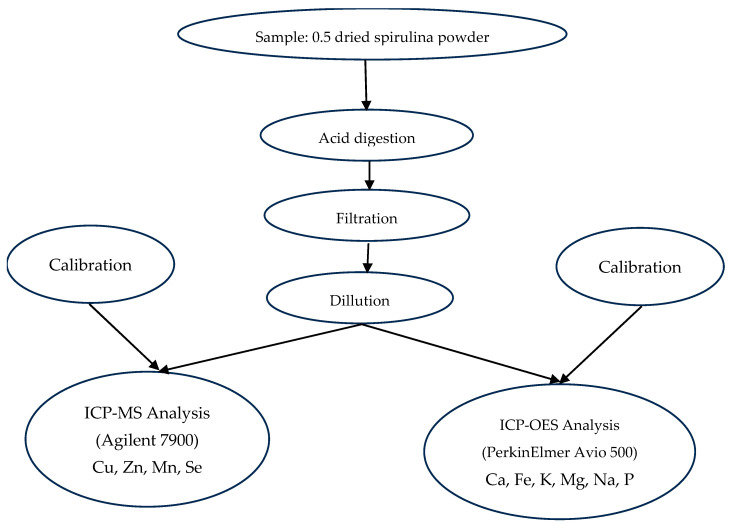
Workflow of mineral element analysis by ICP-OES and ICP-MS.

Mineral composition of *Arthrospira platensis* var. *toliarensis* powder was determined following a two-step procedure: acid digestion according to MSZ EN 13805:2015 [19], followed by elemental quantification using Inductively Coupled Plasma Optical Emission Spectrometry (ICP-OES) and Inductively Coupled Plasma Mass Spectrometry (ICP-MS), depending on element abundance. Trace elements (Cu, Zn, and Se) were measured using ICP-MS (EPA 6020B:2014) [20] due to their low concentrations and the higher sensitivity of this method. Major and abundant elements (Ca, Fe, K, Mg, Na, P, and Mn) were quantified by ICP-OES (EPA 6010D:2018) [21], which is suitable for higher concentrations (Figure 3).

For each sample, 0.5 g of dried spirulina powder was digested in 5 mL of concentrated nitric acid in Teflon vessels using a microwave digestion system (Milestone Ethos UP), with a ramp to 180 °C in 10 min, followed by 20 min at constant temperature. Digests were filtered through 0.45 µm PTFE membranes and diluted to 25 mL with ultrapure water. ICP-MS measurements were performed using an Agilent 7900 system equipped with a concentric nebulizer, quartz torch, and quadrupole analyzer. External calibration was achieved using multielement standards (Inorganic Ventures, IV-STOCK-21, 100 mg/L), diluted to 1–100 µg/L in 2% HNO_3_. An internal standard (Sc, 10 µg/L) was continuously introduced to correct for matrix effects and signal drift. The method’s LODs were 0.025 µg/L for Cu, 0.033 µg/L for Zn, and 0.015 µg/L for Se, with corresponding LOQs of 0.083, 0.110, and 0.050 µg/L, respectively. The elements quantified included Cu (*m*/*z* 63), Zn (*m*/*z* 66), Mn (*m*/*z* 55), and Se (*m*/*z* 78). Macroelements were analyzed using a PerkinElmer Avio 500 ICP-OES spectrometer in axial view mode. The wavelengths used were Ca (317.933 nm), Fe (259.940 nm), K (766.490 nm), Mg (285.213 nm), Na (589.592 nm), and P (177.495 nm). Calibration was conducted with Merck ICP standards at concentrations of 0.5, 2, 5, 10, and 20 mg/L in 2% HNO_3_. The detection limits for ICP-OES were 0.020 mg/L for Ca, 0.010 mg/L for Fe, and 0.015 mg/L for Mg, with LOQs of 0.067, 0.034, and 0.050 mg/L, respectively. Element concentrations were determined by interpolation from calibration curves and expressed as mg/100 g of dry matter.

#### 2.3.5. Procedures for the Analysis of Bioactive Compounds

##### Extraction Procedure

Three distinct extracts were prepared from *Arthrospira platensis* var. *toliarensis* powder to evaluate its polyphenolic content and antioxidant activity: a crude hydroethanolic extract (70% *v*/*v*), a hexane extract, and a residual aqueous extract. Ultrasound-assisted extraction was applied to 20 g of spirulina powder in 200 mL of 70% ethanol (500 rpm, 24 h), followed by 30 min of ultrasonication at 400 W and 30 °C. The crude extract was filtered and evaporated under vacuum. Liquid–liquid partitioning was then performed by dissolving the hydroethanolic crude extract in 150 mL of distilled water and extracting with 150 mL of hexane in a separatory funnel. Both phases (hexane and aqueous) were collected separately and concentrated. In parallel, direct extraction of polyphenols from raw biomass was also conducted (100 mg/mL in 70% ethanol) to assess total phenolic richness without solvent separation bias.

##### Total Phenolic Content (TPC)

TPC was measured using the Folin–Ciocalteu colorimetric method [22]. Two approaches were used: direct quantification from raw biomass (100 mg/mL in 70% ethanol) and comparative analysis of hydroethanolic, hexane, and aqueous extracts at equal concentrations.

Standard calibration curves were constructed using gallic acid, tannic acid, EGCG, epicatechin, and catechin (10–250 mg/L in methanol). For each assay, 100 µL of extract was mixed with 500 µL of diluted Folin–Ciocalteu reagent (1:10), followed by 400 µL of 7.5% sodium carbonate. After 30 min of incubation in the dark at room temperature, absorbance was read at 750 nm (UV-Vis spectrophotometer, Perkin-Elmer, Waltham, MA, USA). Each sample was measured in triplicate. For direct quantification from raw biomass, the results are expressed in multiple equivalents: TAE (mg/kg of dry matter) and EGCG-E, ECE, GAE, and CE (mg/kg of DM). The polyphenol content in the different extracts was expressed as grams of gallic acid equivalents per 100 g of dry extract (DE) (g GAE/100 g of DE).

##### Total Flavonoid Content (TFC)

TFC was assessed via AlCl_3_ complexation [23]. A flavonoid–aluminum complex forms a measurable yellow chromophore at 415 nm. A calibration curve was constructed using quercetin (0.01% in 70% ethanol) at volumes of 0.1 to 0.5 mL, each mixed with 1 mL of 2.5% AlCl_3_, 2 mL of 10% sodium acetate, and ethanol up to 10 mL. After 30 min at room temperature, absorbance was recorded at 415 nm. Samples were analyzed similarly, replacing ethanol with water in final volume adjustments. The results are expressed as g quercetin equivalents per 100 g of dry extract (g QE/100 g of DE).

##### Antioxidant Activity (DPPH)

Antioxidant activity was evaluated using the DPPH radical scavenging assay adapted to the microplate format [23]. A 0.1 mM DPPH solution (3.94 mg/100 mL methanol) was prepared and stabilized in the dark. Extracts were tested at concentrations from 500 to 15.6 µg/mL. Ascorbic acid was used as a reference standard. In a 96-well plate, 100 µL of extract or standard was mixed with 100 µL of DPPH solution and incubated in the dark for 30 min. Absorbance was measured at 517 nm using a TECAN Infinite M200 Pro reader. Dose–response curves were constructed to determine IC_50_ values (concentration required to inhibit 50% of DPPH radicals). Lower IC_50_ values indicate stronger antioxidant capacity. As a reference, ascorbic acid showed an IC_50_ of 1.87 µg/mL under the same conditions.

#### 2.3.6. Toxicological Analysis: Aflatoxin Quantification (B_1_, B_2_, G_1_, and G_2_)

The presence of aflatoxins (B_1_, B_2_, G_1_, and G_2_) in *Arthrospira platensis* var. *toliarensis* powder was evaluated using high-performance liquid chromatography with post-column derivatization and fluorescence detection (HPLC-FLD), as described by Schincaglia et al. (2023) and Yu et al. (2019) [24,25].

Aflatoxins were extracted from 50 g of spirulina powder with 200 mL of methanol/water (80:20, *v*/*v*), homogenized for 3 min, and filtered. A 10 mL aliquot of the filtrate was diluted with 40 mL ultrapure water and then passed through an aflatoxin-specific immunoaffinity column (flow rate: 1 mL/min). The column was washed with 10 mL of water, and aflatoxins were eluted with 1.5 mL of methanol. The eluate was evaporated under nitrogen at 40 °C and reconstituted in 200 µL of methanol. Post-column bromination was performed online using pyridinium hydrobromide and acetic acid. A 100 µL aliquot was injected into an HPLC system equipped with a C18 column. The mobile phase consisted of methanol/water/acetic acid (55:40:5, *v*/*v*/*v*) at 1.0 mL/min, with the column temperature maintained at 35 °C. Fluorescence detection was performed at 365 nm excitation and 435 nm emission. Quantification was achieved using external calibration with aflatoxin standards (B_1_, B_2_, G_1_, and G_2_) at concentrations ranging from 0.5 to 20 µg/kg. The results are expressed in micrograms per kilogram of dry spirulina powder (µg/kg of DM). The method’s limit of detection (LOD) was 0.1 µg/kg, and the limit of quantification (LOQ) was 0.3 µg/kg for all four aflatoxins.

### 2.4. Statistical Analysis

All measurements were performed in triplicate to ensure reproducibility. The results are presented as mean ± standard deviation. Statistical analysis was conducted using Microsoft Excel and Minitab version 9.

Differences between groups were assessed using one-way analysis of variance (ANOVA). A *p*-value < 0.05 was considered statistically significant, indicating rejection of the null hypothesis with a confidence level of 95%

## 3. Results

### 3.1. Experimental Cultivation 

#### 3.1.1. Growth Kinetics of *Arthrospira platensis* var. *toliarensis*

The growth of *Arthrospira platensis* var. *toliarensis* was monitored over a 10-day cultivation period in a semi-artisanal basin (25 L volume, 0.125 m^2^ surface). Biomass development was assessed using two complementary approaches: (a) turbidity measurements with a Secchi disk, converted into dry biomass concentration (g/L), and (b) microscopic counting of the average number of coils (helical trichomes) in two culture drops.

**Figure 4:**
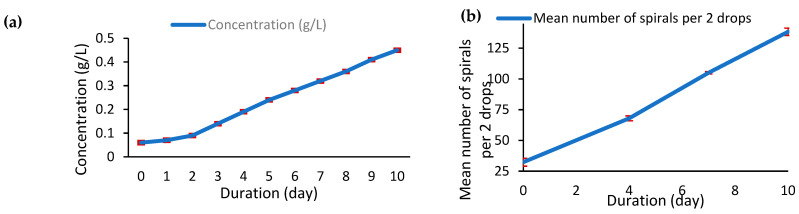
(**a**) Evolution of dry biomass concentration (g/L), (**b**) spire count (coils/2 drops) of *A. platensis* var. *toliarensis* over 10 days of cultivation.

As shown in Figure 4a, the culture exhibited a consistent and substantial increase in biomass throughout the 10 days. Biomass concentration rose from 0.06 ± 0.0012 g/L at Day 0 to 0.45 ± 0.028 g/L by Day 10, representing a 7.5-fold increase. The growth curve delineates two distinct phases. The initial lag phase (Day 0–2) was characterized by minimal biomass accumulation and a shallow slope, suggesting adaptation of the strain to the cultivation environment. From Day 2 onwards, the culture entered an exponential growth phase, with a steady and linear increase in biomass concentration. No plateau or decline was observed by Day 10, indicating that the culture was still in active expansion. The average specific growth rate (μ) during this exponential phase (Day 2–10) was calculated as 0.201 ± 0.0067 day^−1^, confirming the high proliferation potential under the tested conditions. No significant differences were observed between the three treatments (*p* > 0.05).

In parallel, in Figure 4b, microscopic cell counts revealed a proportional increase in cellular density. The number of coils per two drops rose from 32.33 ± 3.21 at Day 0 to 68 ± 2 by Day 4 and 105 ± 1 by Day 7 and peaked at 138.33 ± 3.05 by Day 10. This continuous rise illustrates a dynamic and healthy growth environment without stagnation. Notably, a sharper increase in spire density was observed from Day 4 onward, with a marked surge between Days 7 and 10, further supporting the entry into an advanced exponential phase. Together, macroscopic (turbidity/biomass) and microscopic (coil count) indicators corroborate the vigorous development of the culture throughout the entire experiment.

#### 3.1.2. Biomass and Protein Yield

The final biomass output after 10 days of cultivation was 9.75 ± 0.72 g of dry weight, corresponding to a daily areal productivity of 7.8 ± 0.58 g/m^2^/day. Based on an average protein content of 60% on a dry weight basis, the protein yield was 5.85 ± 0.43 g, equating to a protein productivity of 4.68 ± 0.35 g/m^2^/day (Table 1).

To contextualize the productive potential of *A. platensis* var. *toliarensis* as a sustainable protein source, a comparative assessment with conventional protein systems is presented in Table 1. The spirulina strain used in this study shows higher daily productivity and shorter cultivation time compared to soybean and beef, with a potential for multiple harvests per year. This highlights the considerable efficiency of microalgal biomass production in semi-artisanal systems, particularly under arid and resource-limited settings.

### 3.2. Proximate Composition of Arthrospira platensis var. toliarensis Powder

The biochemical composition of *Arthrospira platensis* var. *toliarensis* harvested during two distinct seasons, May (cool season) and October (hot season), was evaluated to investigate the impact of seasonal environmental conditions on its macronutrient profile. Concentrations are expressed in grams per 100 g of wet weight (WW), and the results are summarized in Table 2.

Moisture content remained low and statistically unchanged between seasons (7.2 ± 0.11% in May vs. 6.9 ± 0.20% in October; *p* > 0.05), indicating consistent post-harvest drying conditions. The corresponding dry matter values were 92.8% and 93.1%, providing a robust basis for comparison. Ash content, which reflects the total mineral fraction, was also stable across seasons (9.15 ± 0.15% vs. 9.32 ± 0.15%; *p* > 0.05), suggesting no major seasonal influence on mineral accumulation. In contrast, protein content showed a significant seasonal increase, rising from 44.61 ± 0.26% in May to 65.12 ± 0.62% in October (*p* ≤ 0.05), which translates to 48.07% and 69.93% on a dry matter basis, respectively. This indicates a favorable protein biosynthesis under warmer and sunnier conditions. Lipid content remained low in both harvests, with a modest, non-significant increase from 1.05 ± 0.39% to 1.75 ± 0.37% (*p* > 0.05), equivalent to 1.13% and 1.88% on a dry weight basis. Interestingly, the total sugar content displayed an opposite trend to proteins: the May harvest contained significantly more sugars (37.98 ± 0.73%) than the October harvest (16.90 ± 1.19%) (*p* ≤ 0.05), corresponding to 40.93% and 18.15% dry matter, respectively. These observations suggest a seasonal shift in metabolic allocation between protein and carbohydrate fractions, likely driven by temperature and light intensity, favoring protein accumulation in hotter conditions.

### 3.3. Micronutrient Composition: Amino Acid and Mineral Profiles

The micronutrient composition of *Arthrospira platensis* var. *toliarensis* was evaluated through amino acid profiling by HPLC and elemental analysis using ICP-OES and ICP-MS, based on samples harvested during two contrasting seasons: the cool season (May) and the hot season (October). The results are presented in Table 3, expressed per 100 g of dry biomass.

Overall, a general increase in amino acid concentrations was observed during the hot season (October), which is consistent with the higher total protein content previously reported for this period. Among the most abundant amino acids in both seasons were glutamic acid, aspartic acid, leucine, and valine. In the hot season, these reached concentrations of 9.23 ± 0.16, 5.87 ± 0.13, 6.10 ± 0.14, and 4.05 ± 0.13 g/100 g of DM, respectively, forming the dominant contributors to the protein fraction of the biomass.

Several essential amino acids, including isoleucine (3.66 ± 0.09 g/100 g of DM), phenylalanine (2.14 ± 0.05 g/100 g of DM), methionine (0.898 ± 0.020 g/100 g of DM), threonine (2.34 ± 0.12 g/100 g of DM), and tyrosine (2.90 ± 0.10 g/100 g of DM), also showed significant increases during the hot season. This suggests a qualitative enhancement of the amino acid profile under warmer and sunnier environmental conditions. In contrast, lysine exhibited relatively stable concentrations across both seasons, with no statistically significant variation.

A few amino acids, notably alanine (from 3.41 to 3.28 g/100 g of DM), glycine (2.13 to 1.92 g/100 g of DM), and cysteine + cystine (0.399 to 0.376 g/100 g of DM), displayed modest reductions in the hot season. Although these decreases were minor, they contrast with the general upward trend observed for most other amino acids. Serine and lysine showed comparable levels between seasons, while proline remained nearly constant at around 1.7 g/100 g of DM. Histidine, however, exhibited a substantial and statistically significant increase from 0.646 to 0.985 g/100 g of DM in the hot season. Hydroxyproline and ornithine were consistently below the limit of quantification in both seasons, which aligns with their typically low representation in cyanobacterial protein matrices.

The mineral profile of *A. platensis* var. *toliarensis* also demonstrated clear seasonal variation. Sodium exhibited the most pronounced change, with a sharp decline from 2140.0 ± 35.4 mg/100 g of DM in the cool season to 687.2 ± 12.3 mg/100 g of DM in the hot season. Similar trends were observed for other trace minerals such as iron (235.0 ± 9.1 to 65.74 ± 1.1 mg/100 g of DM), zinc (12.4 ± 1.3 to 2.5 ± 0.4 mg/100 g of DM), and copper (1.4 ± 0.1 to 0.5 ± 0.1 mg/100 g of DM), which were all significantly more concentrated in the May harvest. These differences may reflect changes in bioavailability and uptake of elements influenced by temperature, salinity, and evaporation rates in open-air systems.

Conversely, the hot-season biomass was enriched in several macro- and microelements. Calcium increased markedly from 580.0 ± 12.1 to 968.0 ± 15.1 mg/100 g of DM, while magnesium nearly doubled (187.0 ± 8.2 to 389.28 ± 6.4 mg/100 g of DM). Phosphorus and manganese also rose significantly, with concentrations of 815.0 ± 13.2 and 3.26 ± 0.6 mg/100 g of DM in the hot season compared to 690.0 ± 14.7 and 1.7 ± 0.2 mg/100 g of DM in the cool season, respectively. Selenium levels remained low across both seasons (0.06–0.08 mg/100 g of DM), with no significant difference, although the absolute value was slightly higher in the October harvest.

When considering the overall mineral abundance, sodium emerged as the dominant element in both seasons, reaching 2140.0 mg/100 g of DM in the cool season. It was followed by potassium (up to 1530.0 mg/100 g of DM), phosphorus (815.0 mg/100 g of DM), calcium (968.0 mg/100 g of DM), and magnesium (389.28 mg/100 g of DM), which together define the core macroelement composition of this strain. These electrolytes are essential for maintaining physiological functions and confer added value to *Arthrospira* as a functional food. On the other hand, selenium (≤0.08 mg/100 g of DM), copper (≤1.4 mg/100 g of DM), manganese (≤3.26 mg/100 g of DM), and zinc (≤12.4 mg/100 g of DM) were present in lower quantities yet play vital roles in enzymatic catalysis, antioxidant protection, and cellular regulation. The seasonal modulation of these micronutrients underlines the need to consider environmental timing when cultivating *Arthrospira* for targeted nutritional applications.

### 3.4. Analysis of Bioactive Compounds

To evaluate the phytochemical richness and antioxidant potential of *Arthrospira platensis* var. *toliarensis*, several bioactive compound classes were analyzed, including total polyphenols (TPC) and total flavonoids (TFC), along with their associated radical scavenging activity assessed by DPPH assay. Analyses were conducted both directly on the dry biomass and on solvent-based extracts (hydroethanolic, hexanic, and aqueous), allowing for a comparative evaluation of the chemical profile as influenced by polarity.

#### 3.4.1. Total Polyphenol Content in Dry Biomass

The total polyphenol content in the dry biomass was quantified using multiple calibration standards to better capture the structural diversity of phenolic constituents (Table 4). The results reveal values ranging from 1720 to 4110 mg/kg of dry biomass depending on the standard used. The highest polyphenol concentration was observed with EGCG equivalence (4110 ± 0.03 mg/kg of DM), followed by tannic acid (3410 ± 0.11 mg/kg of DM), gallic acid (2830 ± 0.03 mg/kg of DM), catechin (2260 ± 0.04 mg/kg of DM), and epicatechin (1720 ± 0.05 mg/kg of DM). These differences highlight the variability in absorbance and molecular mass of the reference compounds and suggest a chemically diverse phenolic profile in the biomass.

#### 3.4.2. Polyphenols, Flavonoids, and Antioxidant Activity in Solvent Extracts

The quantification of TPC and TFC in the hydroethanolic, hexanic, and aqueous extracts showed marked variations based on the solvent used (Table 5). The hydroethanolic extract exhibited the highest concentration of polyphenols (4.67 ± 0.062 g GAE/100 g of dry extract (DE)), followed by the aqueous (3.77 ± 0.027 g) and hexanic (1.61 ± 0.074 g) extracts. A similar trend was observed for flavonoid content, with the hydroethanolic extract again showing the highest level (2.05 ± 0.036 g QE/100 g of DE), followed by the aqueous (1.65 ± 0.08 g QE/100 g of DE) and hexanic (1.405 ± 0.036 g QE/100 g of DE) extracts. These results suggest that polar solvents, such as ethanol and water, are more effective for extracting both polyphenols and flavonoids, although some lipophilic flavonoid compounds may still be partially recovered by less polar solvents.

Antioxidant capacity, evaluated via the DPPH radical scavenging assay, further supported the distinct bioactive potential of each extract. The hexanic extract displayed the strongest antioxidant activity, with an IC_50_ value of 101.03 ± 1.37 µg/mL, compared to 213.77 ± 2.15 µg/mL for the hydroethanolic extract (Table 5). Although both values remain significantly higher than that of pure ascorbic acid (1.87 ± 0.04 µg/mL), they nonetheless indicate a relevant antioxidant potential, especially attributable to lipophilic components, such as carotenoids, tocopherols, and unsaturated fatty acids in the hexanic phase. The aqueous extract was not tested in this assay.

### 3.5. Toxicological Analysis: Aflatoxin Quantification

The toxicological safety of *Arthrospira platensis* var. *toliarensis* was assessed through the quantification of four major aflatoxins, B1, B2, G1, and G2, in samples harvested during two contrasting periods: the cool season (May) and the hot season (October). The results are presented in Table 6.

The measured concentrations of aflatoxins were consistently low across both sampling periods. In the cool-season biomass, aflatoxins G1 and B1 were each detected at 0.1 µg/kg of DM, while G2 and B2 remained below the limit of quantification (<0.1 µg/kg of DM), resulting in a total aflatoxin content of 0.2 µg/kg of DM. During the hot season, B1 concentration slightly increased to 0.2 µg/kg of DM, bringing the total aflatoxin level to 0.3 µg/kg of DM. However, these variations are minor and not statistically significant.

Overall, the results demonstrate that *A. platensis* var. *toliarensis* contains only trace amounts of aflatoxins, which are well below regulatory thresholds for food and dietary supplement safety.

## 4. Discussion

### 4.1. Cultivation Performance of Arthrospira platensis *var.* toliarensis

The experimental cultivation of *Arthrospira platensis* var. *toliarensis* under a small-scale low-input outdoor or semi-artisanal system simulating smallholder practices demonstrated a sustained growth pattern and promising yields, confirming the adaptability of this endemic strain to local environmental conditions. The observed growth curve featured a distinct exponential phase, with a maximum dry biomass concentration of 0.45 g/L achieved in 10 days, corresponding to a cumulative yield of 9.75 ± 0.72 g. The calculated areal productivity of 7.8 ± 0.58 g/m^2^/day places this result within the upper range reported for artisanal systems (typically 4–7 g/m^2^/day), approaching the yields of semi-intensive cultivation methods or optimized intensive systems in photobioreactors or raceways enriched with CO_2_ (10–20 g/m^2^/day) [33,34,35].

The physicochemical conditions of the culture medium, daily temperatures ranging between 22 °C and 29 °C, a pH between 8.5 and 8.72, and a moderately increasing salinity, proved to be well-suited to the physiological requirements of *A. platensis*. These findings align with previous studies [36,37], which report optimal growth within 25–35 °C and an alkaline pH between 8.5 and 10.5.

Microscopic cell counts (ranging from 32 to 138 spirals) corroborated the macroscopic growth observations. The use of simple monitoring tools, such as a Secchi disk and gravimetric biomass measurements, highlights the feasibility of reproducible cultivation under low-tech conditions. This approach, while technically simplified, enables scientifically rigorous production practices without the need for advanced infrastructure, thus supporting the resilience of decentralized food systems.

The protein yield, estimated at 5.85 ± 0.43 g over 0.125 m^2^ in 10 days (equivalent to 4.68 ± 0.26 g protein/m^2^/day), demonstrates the competitive potential of *A. platensis* var. *toliarensis* compared to conventional plant-based protein crops. For instance, soybeans, despite their high protein content (35.35–40.30%, [38]), typically require 90 to 150 days to produce between 12 and 170 g protein/m^2^ [26,28,29]. In terms of water-use efficiency, spirulina cultivation (2200 L/kg) is on par with soybean production (2145 L/kg; [27]) but benefits from a shorter production cycle and the potential for culture medium reuse. In contrast, beef production exhibits extremely low protein productivity per area (0.00074 g/m^2^/day) and a substantially higher water footprint (>15,000 L/kg) while also generating average greenhouse gas emissions of 105 kg CO_2_-eq per 100 g of protein [30,39]. In this context, spirulina emerges as a high-yielding, resource-efficient, and environmentally sustainable alternative that is particularly relevant for arid or resource-limited regions such as southern Madagascar.

### 4.2. Biochemical Composition and Nutritional Value

A comparative analysis of the biochemical and mineral composition of *Arthrospira platensis* var. *toliarensis* harvested during two distinct seasons reveals marked seasonal variability, emphasizing the influence of climatic conditions on the accumulation of key biomolecules. This metabolic adaptability to environmental fluctuations, frequently documented in cyanobacteria, is well supported in the recent literature [40,41] and provides *A. platensis* var. *toliarensis* with a strategic advantage for sustainable local cultivation, particularly in tropical regions such as southern Madagascar, where production conditions can vary throughout the year.

Moisture content is a critical parameter for product quality, microbiological safety, shelf stability, and the commercial value of spirulina powder. In this study, the moisture levels of *A. platensis* var. *toliarensis* samples were 7.2 ± 0.11% in May and 6.9 ± 0.20% in October, with no statistically significant seasonal difference (*p* > 0.05). These values remain below the recommended threshold of 7–11% for commercial spirulina powders [42], indicating an effective dehydration process in line with industry quality standards. Low moisture content is essential to minimize microbial growth and limit enzymatic or oxidative degradation, thereby preserving product stability.

Protein content, which is significantly higher during the hot season (65.12%, equivalent to 69.93% on a dry matter basis) compared to the cool season (44.61%), emerged as one of the most nutritionally relevant findings. While this concentration falls within the typical range reported for *Arthrospira* strains (50–70% of DM; [43,44]), it nonetheless underscores the exceptional protein richness of the *toliarensis* variety. This seasonal variation likely results from increased temperature and light intensity in October, which enhance photosynthetic activity and the synthesis of structural proteins [45,46]. Vonshak (1997) [37] also demonstrated that harvesting during the exponential or late stationary growth phase, particularly under elevated temperatures (>25 °C), results in higher protein accumulation. This enrichment is highly relevant for the formulation of protein-dense therapeutic foods such as RUTFs, where protein quality and quantity are critical criteria.

When compared to major dietary protein sources such as beef (17.4–22%), poultry (19–22%), fish (19.2–22%), and soybeans (35.35–40.30%) [38], *A. platensis* var. *toliarensis* stands out as one of the richest known protein sources. This potential is further reinforced by the abundance and quality of its amino acid profile. Glutamic acid, leucine, aspartic acid, and valine were the dominant constituents, consistent with observations in other *A. platensis* strains and cultivation systems [47,48]. The significant increase in essential amino acids (EAAs), particularly isoleucine, phenylalanine, and methionine, further enhances the nutritional value of biomass harvested in the hot season. Notably, methionine, often limited in microalgae, reached ~0.9 g/100 g of DM, surpassing levels typically found in conventional plant proteins such as soybeans [49] or chickpeas [50]. Although tryptophan was not quantified due to its degradation during acid hydrolysis or analytical limitations, the presence of most EAAs in balanced proportions supports the suitability of this biomass for treating protein-energy malnutrition, especially in children.

Lipid content ranged from 1.05 ± 0.39% to 1.75 ± 0.37% (fresh weight), equivalent to 1.13–1.88% on a dry matter basis, with values lower than the typical 5–14% of DM reported in the literature for *A. platensis* [38,51]. This low lipid content may reflect a metabolic orientation favoring the synthesis of structural and functional macromolecules such as proteins and pigments over energy storage lipids. This trend was also reported by Moreno-Garcia et al. (2019) [52], who found that lipid content and composition in microalgae are strongly modulated by environmental factors, influencing resource allocation patterns during cultivation. Despite its modest quantity, the lipid fraction remains nutritionally relevant due to its composition, which includes polyunsaturated fatty acids, such as γ-linolenic acid (GLA), linoleic acid, and palmitic acid compounds, with documented anti-inflammatory, cardioprotective, and immunomodulatory effects [51,53,54]. Even in low doses, these lipids contribute meaningfully to the functional quality of dietary supplements and enriched foods.

The observed decrease in sugar content in hot-season samples could be attributed to a metabolic reallocation of carbon in response to heat stress. Under long exposure (more than 8 h) to elevated temperatures, cyanobacteria, such as *Arthrospira*, may redirect carbon flux toward the synthesis of proteins and lipids, particularly those involved in thermal protection, including genes for heat shock proteins, at the expense of carbohydrate reserves [55]. Previous outdoor cultivation studies have confirmed that carbohydrate accumulation in *Spirulina platensis* is significantly higher at suboptimal temperatures (e.g., around 25 °C), while elevated summer temperatures (~35 °C) are associated with reduced polysaccharide levels, likely due to carbon redirection into alternative metabolic pathways [56].

As in other *Arthrospira* strains [43,48,57,58], the mineral profile of *A. platensis* var. *toliarensis* confirms its status as one of the most nutrient-dense microorganisms known, particularly in the context of malnutrition. The analysis reveals high concentrations of macroelements (Na, K, Ca, Mg, and P) and functional trace elements (Fe, Zn, Mn, and Se), conferring exceptional nutritional value to this cyanobacterium. Calcium (up to 968 ± 15.1 mg/100 g of DM), magnesium (389 ± 6.4 mg/100 g of DM), and phosphorus (815 ± 13.2 mg/100 g of DM) were present at levels significantly higher than those typically found in leafy vegetables or cereals [59,60]. Notably, iron content reached 235 ± 9.1 mg/100 g of DM under certain conditions, far exceeding the concentrations in iron-rich foods, such as legumes (1.3–2.3 mg/100 g; [61]) or *Moringa oleifera* leaf powder (25.6–28.2 mg/100 g; [62]). This iron, largely bound to transport or storage proteins, is more bioavailable than non-heme plant iron, making *A. platensis* particularly relevant for anemia prevention and treatment [63,64]. The presence of zinc (up to 12.4 ± 12.4 mg/100 g of DM) and copper (1.4 ± 0.1 mg/100 g of DM) complements this micronutrient profile essential for growth, immunity, and cellular development [65]. Selenium, though present in modest amounts (0.06–0.08 mg/100 g of DM), is sufficient to meet daily requirements with less than 10 g of dried spirulina while contributing to antioxidant defense mechanisms [66] (Castel et al., 2024).

Despite its overall mineral richness, *Arthrospira platensis* var. *toliarensis* exhibited marked seasonal variability in elemental composition, reflecting the influence of environmental conditions on mineral uptake dynamics.

### 4.3. Phytochemical Richness and Antioxidant Activity

The total polyphenol content of *Arthrospira platensis* var. *toliarensis*, determined directly from the dried biomass, reached values as high as 4110 mg/kg of DM in EGCG equivalents and 2830 mg/kg in gallic acid equivalents. These substantial concentrations highlight the microalga’s potential as a valuable ingredient in functional food applications, given the well-established antioxidant, anti-inflammatory, and lipid peroxidation-inhibitory properties of polyphenols [67,68]. Among the solvent extracts tested, the hydroethanolic extract exhibited the highest polyphenol content (4.67 g GAE/100 g of DE), followed by the aqueous (3.77 g GAE/100 g of DE) and hexanic extracts (1.61 g GAE/100 g of DE). This extraction hierarchy corroborates findings by Rungjiraphirat et al. (2025) [69], who also reported maximal recovery of polyphenols from *A. platensis* using ethanol compared to more polar (water) or less polar (methanol) solvents. Notably, the polyphenol concentrations observed in this study surpass previously reported values, which averaged 38.8 mg GAE/g of DE, reaching up to 46.7 mg GAE/g. These findings indicate that the majority of polyphenols in *A. platensis* var. *toliarensis* exhibit intermediate-to-low polarity, thus supporting the use of ethanol-based or ethanol–water extraction systems for optimal recovery. Despite its lower overall polyphenol yield, the hexanic extract retained a noteworthy amount of phenolic compounds, suggesting the presence of amphiphilic or lipophilic molecules such as tocopherols, hydroxylated carotenoids, or phenol-lipids, which are preferentially extracted by non-polar solvents [70].

Flavonoid quantification followed a comparable trend. The hydroethanolic extract yielded the highest flavonoid content (2.05 g QE/100 g of DE), followed by the aqueous (1.65 g QE/100 g of DE) and hexanic extracts (1.41 g QE/100 g of DE). This distribution suggests that the flavonoids present in *A. platensis* var. *toliarensis* are predominantly semi-polar, with a fraction of less polar or lipophilic compounds also extractable in hexane. Although slightly lower than concentrations reported in other studies [71,72], the flavonoid content observed in this study remains nutritionally relevant. These compounds contribute to intracellular oxidative regulation and exhibit potential bioactivities, including anti-inflammatory, radical-scavenging, and antimicrobial effects.

The antioxidant activity, assessed through the DPPH radical scavenging assay, demonstrated moderate efficacy across all extracts. The hexanic extract exhibited the strongest activity (IC_50_ = 101.03 µg/mL), nearly twice as potent as the hydroethanolic extract (IC_50_ = 213.77 µg/mL), although both were substantially less active than the positive control, ascorbic acid (IC_50_ = 1.87 µg/mL). These results align with the chemical composition of each extract: the hexanic fraction is enriched in lipophilic antioxidants such as carotenoids, tocopherols, and unsaturated fatty acids, all of which are known to efficiently stabilize lipid peroxyl radicals [48,73,74]. In contrast, the hydroethanolic extract primarily contains hydrophilic phenolic compounds, which exhibit weaker reactivity in DPPH assays but may exert superior antioxidant effects in biological systems such as plasma, cellular membranes, and cytosolic compartments. Despite the strong DPPH activity of the hexanic extract, the bioavailability of lipophilic antioxidants such as carotenoids and tocopherols in humans can be limited due to their dependence on lipid digestion and micellar incorporation. These compounds are less soluble in aqueous environments and require dietary fats for optimal absorption. Once absorbed, they tend to accumulate in lipid-rich tissues, providing localized protection against oxidative damage rather than systemic effects [75,76].

Compared to standard *Arthrospira platensis* strains commonly cultivated in Asia and Europe, *A. platensis* var. *toliarensis* displayed comparable and, in some cases, superior nutritional properties under low-input cultivation. For instance, the protein content observed in the cold-season harvest exceeded average values reported in commercial powders (typically 55–70 g/100 g of DM) [43,44]. Similarly, the mineral content (e.g., Fe, Ca, and Mg) was notably higher than that of *Spirulina platensis* cultivated in photobioreactors, suggesting a potential advantage of the Toliara strain for micronutrient-rich supplementation [57]. Furthermore, the strong antioxidant capacity observed in hexanic extracts (IC_50_ = 101 µg/mL) highlights the presence of lipophilic antioxidants, such as carotenoids and tocopherols, which have also been reported in *A. platensis* but are typically more abundant under environmental stress conditions that this endemic strain endures naturally. These results support the unique adaptive and functional potential of *A. platensis* var. *toliarensis* under artisanal, open-pond systems, contrasting with the tightly controlled growth conditions required for standard strains

### 4.4. Toxicological Safety

Toxicological screening of *A. platensis* var. *toliarensis* reveals consistently low levels of aflatoxins B1, B2, G1, and G2, with total concentrations of 0.2 µg/kg of DM in the cool season and 0.3 µg/kg of DM in the hot season. These values remain well below international regulatory thresholds for food-grade powders, indicating a high degree of safety for human consumption. Following European legislation, Regulation (EU) 2023/915 [77], the maximum permitted limit for total aflatoxins (B1, B2, G1, and G2) in similar food matrices is 5 µg/kg, with a stricter threshold of 2 µg/kg for aflatoxin B1 in certain processed foods. Across several African countries, including Nigeria, South Africa, and Kenya, national limits for aflatoxins range from 5 to 20 µg/kg depending on the specific food product [78]. To date, Madagascar lacks national standards regarding aflatoxin contamination. Nonetheless, the values reported here demonstrate compliance with international norms, underscoring the microbial and toxicological safety of this biomass.

It is important to note that cyanobacteria do not inherently produce aflatoxins, which are mycotoxins primarily synthesized by fungal species belonging to the genus *Aspergillus*, notably *A. flavus* and *A. parasiticus* [79]. Contamination is typically exogenous and may occur during pre- or post-harvest phases, depending on environmental conditions such as temperature, humidity, water stress, substrate characteristics, pH, hygiene during cultivation, processing, and storage [80]. The exceptionally low levels of aflatoxins found in this study strongly suggest that the spirulina biomass was handled under controlled and hygienic conditions, particularly during the harvest and drying phases. Maintaining such practices is essential to mitigate fungal contamination, especially during the hot season when moisture and temperature levels pose greater risks.

Recent evidence also highlights the capacity of spirulina not only to resist aflatoxin contamination but also to exert protective effects in animal models exposed to these mycotoxins. Studies have reported that spirulina matrices can attenuate aflatoxin-induced hepatotoxicity, likely due to their antioxidant and membrane-stabilizing properties [81,82]. While such functional benefits do not substitute for rigorous compliance with safety regulations, they enhance the nutritional and therapeutic relevance of *A. platensis* in environments where trace contamination remains a potential concern.

Although aflatoxin levels in the analyzed batch were well below international safety thresholds, it is important to note that batch-to-batch variability may occur due to environmental or post-harvest factors. Therefore, routine monitoring of aflatoxin contamination is recommended to ensure consistent safety across production cycles, especially before use as food or feed.

In addition to the reassuringly low aflatoxin levels observed, it is important to consider other potential safety concerns associated with the consumption of *Arthrospira* species. Although generally regarded as safe by the FDA, cases of allergic reactions, including anaphylaxis, have been reported in sensitive individuals consuming *Spirulina* or *Arthrospira* supplements. These reactions are thought to be linked to specific phycobiliproteins or bioactive peptides present in the biomass [83,84]. Furthermore, consumption of microalgae may be contraindicated in certain populations, such as individuals with phenylketonuria due to the high phenylalanine content or those with autoimmune diseases, where immune stimulation might be undesirable. In addition to potential allergenicity, microalgae may accumulate anti-nutritional factors, notably heavy metals (e.g., arsenic, lead, cadmium, and mercury) and cyanotoxins in contaminated environments [85,86,87]. Therefore, although *Arthrospira platensis* var. *toliarensis* shows promising nutritional and functional properties, regular safety monitoring, including allergenicity and broader toxicological screening, is warranted, especially before its inclusion in vulnerable populations’ diets or therapeutic formulations.

These risks can be mitigated by stringent quality control, regular contaminant monitoring, and cultivation under well-managed, traceable conditions. Adhering to the recommended intake levels, typically 2–10 g per day for healthy adults, up to a maximum of 30 g, is also crucial to avoid potential adverse effects [88,89]. For these reasons, batch-to-batch variability in bioactive and toxic components should be assessed, especially prior to any use as food or feed. We recommend routine screenings for aflatoxins, heavy metals, and microbial contaminants, along with allergenicity assessment in future studies.

### 4.5. Nutritional, Industrial, and Functional Perspectives: Arthrospira platensis var. toliarensis as a Strategic Food-Grade Super-Ingredient

The findings of this study highlight the remarkable nutritional potential of *Arthrospira platensis* var. *toliarensis*, particularly during the hot season. Its high protein content, comparable to, or even surpassing, that of conventional animal- and plant-based protein sources, combined with a stable and balanced mineral composition, positions this microalga as a strategic candidate for the fortification of food products. This is especially relevant for applications aimed at combating malnutrition, such as Ready-to-Use Therapeutic Foods (RUTFs), and for the development of functional nutrition solutions.

From a technological standpoint, its low residual moisture content (<7%) offers a significant advantage by ensuring high microbiological and physicochemical stability during storage. Coupled with its low lipid content, this property minimizes the risk of oxidative degradation and enhances the shelf life of dry formulations without the need for artificial preservatives. These attributes endow *A. platensis* var. *toliarensis* with robust processing stability, making it suitable for integration into local value chains while maintaining product quality and traceability.

The presence of highly functional proteins further enhances its relevance for formulation within the food and nutraceutical industries. These proteins exhibit valuable techno-functional properties such as solubility, water retention, emulsifying capacity, and gel formation [90], making them suitable for incorporation into a wide array of products, including nutritional powders, energy bars, fortified biscuits, and high-value dietary supplements.

In addition, the high density of bioactive compounds, particularly polyphenols and flavonoids, confers added value for the development of antioxidant-enriched functional foods. Liposoluble (hexanic) and hydrosoluble (hydroethanolic) extracts may be leveraged for the production of targeted nutritional formulations, such as fortified powders, encapsulated extracts, improved porridges, or matrix-integrated antioxidant carriers. Specifically, hexanic extracts may be beneficial in addressing the needs of children suffering from acute malnutrition through their provision of lipid-soluble antioxidants and membrane-stabilizing agents. Ethanol-based extracts, which are food-grade and more versatile, may be employed in the formulation of liquid or semi-solid preparations or as enriching additives in staple foods.

Finally, future research should focus on the precise identification and characterization of the active biomolecules, such as pigments, bioactive peptides, and phenolic acids, through advanced chromatographic and spectrometric techniques. Such efforts would allow for the functional optimization of spirulina biomass and broaden its therapeutic and nutritional applications, reinforcing its status as a high-value local resource for sustainable food systems.

## 5. Conclusions

This study represents the first comprehensive characterization of *Arthrospira platensis* var. *toliarensis*, a locally cultivated microalga endemic to Madagascar, under semi-artisanal conditions. The findings demonstrate that this strain exhibits robust growth kinetics, high protein productivity, and rich nutritional profiles, particularly during the hot season. With protein content exceeding 65% and essential amino acid levels that rival or surpass conventional sources, such as soybean and fish, *A. platensis* var. *toliarensis* emerges as a highly valuable resource for addressing protein-energy malnutrition.

Mineral analyses reveal substantial concentrations of key micronutrients, including iron, calcium, and magnesium, supporting its relevance for micronutrient fortification strategies. Moreover, the presence of bioactive compounds, namely polyphenols and flavonoids, confers antioxidant properties that further enhance its value as a functional food ingredient. Importantly, toxicological assessment confirms that aflatoxin levels were consistently below international safety thresholds, ensuring the microbiological quality and safety of the biomass.

The combination of high nutritional density, low environmental footprint, and resilience under low-input cultivation systems positions *A. platensis* var. *toliarensis* as a strategic candidate for integration into sustainable food systems. This is particularly relevant for regions facing structural food insecurity and climate vulnerability, such as the Global South. Further research focusing on compound-specific profiling and functional applications in food formulations will help unlock the full potential of this endemic cyanobacterium as a super-ingredient for next-generation nutrition solutions.

## Figures and Tables

**Table 1:** Comparative productivity and resource efficiency of selected protein sources.

Protein Source	Daily Productivity (g/m^2^/day)	Protein Yield (g/m^2^/day)	Production Duration (days)	References
Spirulina (this study)	7.8 ± 0.58	4.68 ± 0.35	10 (up to 300/year)	Present study
Soybean (grain)	33–421 (seasonal)	12–170 (annual)	90–150	[26,27,28,29]
Beef (ruminant meat)	0.37–11.9 (extensive systems)	~0.00074	600–900	[30,31,32]

**Table 2:** Proximate composition (g/100 g of wet weight (WW)) of *A. platensis* var. *toliarensis* harvested in different seasons.

Macronutrient	Cool Season (May)	Hot Season (October)
Moisture (%)	7.2 ± 0.11 ᵃ	6.9 ± 0.20 ᵃ
Ash (%)	9.15 ± 0.15 ᵃ	9.32 ± 0.15 ᵃ
Crude Protein (%)	44.61 ± 0.26 ᵇ	65.12 ± 0.62 ᵃ
Lipids (%)	1.05 ± 0.39 ᵃ	1.75 ± 0.37 ᵃ
Total Sugars (%)	37.98 ± 0.73 ᵃ	16.90 ± 1.19 ᵇ

Means with different superscript letters in the same row are significantly different (*p* ≤ 0.05).

**Table 3:** Amino acid and mineral composition (per 100 g of dry matter (DM)) of *A. platensis* var. *toliarensis* harvested in two different seasons.

Component	Cool Season (May)	Hot Season (October)
**Amino Acids (g/100 g of (DM))**		
Alanine	3.41 ± 0.05 ᵃ	3.28 ± 0.06 ᵇ
Arginine	2.72 ± 0.04 ᵇ	3.91 ± 0.08 ᵃ
Aspartic acid	4.32 ± 0.06 ᵇ	5.87 ± 0.13 ᵃ
Glutamic acid	6.19 ± 0.09 ᵇ	9.23 ± 0.16 ᵃ
Glycine	2.13 ± 0.03 ᵃ	1.92 ± 0.04 ᵇ
Histidine	0.646 ± 0.012 ᵇ	0.985 ± 0.022 ᵃ
Hydroxyproline	<0.2 (LOQ) ᵇ	<0.2 (LOQ) ᵇ
Isoleucine	2.40 ± 0.03 ᵇ	3.66 ± 0.09 ᵃ
Leucine	3.81 ± 0.05 ᵇ	6.10 ± 0.14 ᵃ
Lysine	1.82 ± 0.03 ᵃ	1.75 ± 0.04 ᵃ
Ornithine	<0.05 (LOQ) ᵇ	<0.05 (LOQ) ᵇ
Phenylalanine	1.89 ± 0.03 ᵇ	2.14 ± 0.05 ᵃ
Proline	1.68 ± 0.03 ᵇ	1.71 ± 0.08 ᵇ
Serine	2.08 ± 0.03 ᵃ	2.01 ± 0.06 ᵃ
Threonine	2.12 ± 0.03 ᵃ	2.34 ± 0.12 ᵃ
Tyrosine	1.73 ± 0.02 ᵇ	2.90 ± 0.10 ᵃ
Valine	2.84 ± 0.04 ᵇ	4.05 ± 0.13 ᵃ
Cystine + Cysteine	0.399 ± 0.008 ᵇ	0.376 ± 0.015 ᵇ
Methionine	0.855 ± 0.014 ᵇ	0.898 ± 0.020 ᵃ
**Minerals (mg/100 g of DM)**		
Sodium (Na)	2140.0 ± 35.4 ᵇ	687.2 ± 12.3 ᵃ
Potassium (K)	1530.0 ± 21.8 ᵇ	1228.35 ± 10.4 ᵃ
Calcium (Ca)	580.0 ± 12.1 ᵃ	968.0 ± 15.1 ᵇ
Magnesium (Mg)	187.0 ± 8.2 ᵃ	389.28 ± 6.4 ᵇ
Phosphorus (P)	690.0 ± 14.7 ᵃ	815.0 ± 13.2 ᵇ
Iron (Fe)	235.0 ± 9.1 ᵇ	65.74 ± 1.1 ᵃ
Zinc (Zn)	12.4 ± 1.3 ᵇ	2.5 ± 0.4 ᵃ
Manganese (Mn)	1.7 ± 0.2 ᵃ	3.26 ± 0.6 ᵇ
Copper (Cu)	1.4 ± 0.1 ᵇ	0.5 ± 0.1 ᵃ
Selenium (Se)	0.06 ± 0.01 ᵃ	0.08 ± 0.01 ᵇ

Means with different superscript letters in the same row are significantly different at *p* ≤ 0.05. LOQ: limit of quantification.

**Table 4:** Total polyphenol content in dry biomass of *Arthrospira platensis* var. *toliarensis* using different calibration standards.

Standard Equivalent	Total Polyphenols (mg/kg of DM)
Gallic acid (GAE)	2830 ± 0.03
Tannic acid	3410 ± 0.11
EGCG (epigallocatechin gallate)	4110 ± 0.03
Epicatechin	1720 ± 0.05
Catechin	2260 ± 0.04

Values expressed as mean ± SD.

**Table 5:** TPC, TFC, and antioxidant capacity of different *Arthrospira platensis* var. *toliarensis* extracts.

Extraction Solvent	TPC (g GAE/100 g of DE)	TFC (g QE/100 g of DE)	DPPH IC_50_ (µg/mL)
Hydroethanolic extract	4.67 ± 0.062	2.05 ± 0.036	213.77 ± 2.15
Hexanic extract	1.61 ± 0.074	1.405 ± 0.036	101.03 ± 1.37
Aqueous extract	3.77 ± 0.027	1.65 ± 0.08	–
Ascorbic acid			1.87 ± 0.04

Values expressed as mean ± SD. GAE: gallic acid equivalent; QE: quercetin equivalent; IC_50_: inhibitory concentration 50%.

**Table 6:** Aflatoxin content (µg/kg of dry matter (DM)) in *Arthrospira platensis* var. *toliarensis* harvested in two different seasons.

Aflatoxin	Cool Season (May)	Hot Season (October)
Aflatoxin G1 (µg/kg)	0.1	0.1
Aflatoxin B1 (µg/kg)	0.1	0.2
Aflatoxin G2 (µg/kg)	<0.1	<0.1
Aflatoxin B2 (µg/kg)	<0.1	<0.1
**Total aflatoxins**	**0.2**	**0.3**

## Data Availability

The original contributions presented in this study are included in the article. Further inquiries can be directed to the corresponding author.
